# Stable Isotope Labelling Kinetics (SILK) tracing of neurofilament light in human cerebrospinal fluid and brain tissue

**DOI:** 10.1002/alz.095735

**Published:** 2025-01-09

**Authors:** Tatiana A. Giovannucci, Claire A. Leckey, John Coulton, Henrik Zetterberg, Donald L. Elbert, Randall J. Bateman, Kevin Mills, Selina Wray, Nupur Ghoshal, Chihiro Sato, Ross W. Paterson

**Affiliations:** ^1^ UCL Queen Square Institute of Neurology, London UK; ^2^ UCL Great Ormond Street Institute of Child Health, London UK; ^3^ The Tracy Family SILQ Center, Washington University School of Medicine, St. Louis, MO USA; ^4^ Washington University School of Medicine, St. Louis, MO USA; ^5^ Clinical Neurochemistry Laboratory, Sahlgrenska University Hospital, Mölndal Sweden; ^6^ University of Washington, Seattle, WA USA; ^7^ Darent Valley Hospital, Dartford UK; ^8^ Dementia Research Centre, UCL Queen Square Institute of Neurology, London UK

## Abstract

**Background:**

Neurofilament light protein (NfL) is a promising biomarker of neuronal injury and neurodegeneration. NfL levels in cerebrospinal fluid (CSF) and blood provide information about disease progression and are increasingly relied on as outcome measure in clinical trials. Understanding NfL kinetics *in vivo* is critical for interpreting NfL in response to new events where a steady state cannot be assumed, such as acute injury, disease onset or progression, or response to disease‐modifying therapies.

**Method:**

We infused human participants with diagnosed primary tauopathies (progressive supranuclear palsy, n = 5; corticobasal syndrome, n = 3; behavioural variant frontotemporal dementia, n = 2) with a stable isotope tracer (^13^C_6_‐leucine) and collected CSF by lumbar puncture at 4, 14, 20, 60 and 120 days post‐labelling. In addition, *post‐mortem* brain tissue from three participants who were infused with the tracer 18, 44 and 50 months earlier were homogenised and biochemically fractionated to separate the soluble and insoluble fraction. NfL was enriched in all samples via immunoprecipitation. The ratio of labelled to unlabelled NfL was measured monitoring proteotypic peptides using established targeted mass spectrometry workflows to quantitate the tracer‐to‐tracee ratio (TTR).

**Result:**

Analysis of CSF detected low labelling of NfL (0.04 – 0.36% TTR) by 120 days that was comparable to the TTR levels detected in the soluble brain fraction. There was NfL present in the insoluble fraction (2.87% of the total NfL at 18‐, 8.87% at 44‐ and 14.03% at 50‐months *post‐mortem*, averaged across several NfL peptides), with different relative abundances of NfL domains between these fractions. Interestingly, the ^13^C_6_‐labelled NfL signal detected in the insoluble fraction was more abundant, despite a higher recovery of total NfL in the soluble fraction.

**Conclusion:**

NfL turnover *in vivo* is remarkably slow as it is scarcely captured by 120 days post‐labelling. The labelling results from brain tissue suggest that in these cases of primary tauopathies newly‐synthesized NfL might be delocalized to a pool of protein of low solubility that does not contribute to NfL levels in CSF. Current experiments are addressing whether newly‐synthesized NfL might be sequestered into pathological inclusions.

